# A Real-World Analysis of Immune Checkpoint Inhibitor-Based Therapy After Osimertinib Treatment in Patients With *EGFR*-Mutant NSCLC

**DOI:** 10.1016/j.jtocrr.2022.100388

**Published:** 2022-08-06

**Authors:** Kenji Morimoto, Ryo Sawada, Tadaaki Yamada, Koichi Azuma, Kentaro Ito, Yasuhiro Goto, Hideharu Kimura, Taishi Harada, Shinsuke Shiotsu, Nobuyo Tamiya, Yusuke Chihara, Takayuki Takeda, Osamu Hiranuma, Isao Hasegawa, Yoshie Morimoto, Masahiro Iwasaku, Shinsaku Tokuda, Koichi Takayama

**Affiliations:** a Department of Pulmonary Medicine, Graduate School of Medical Science, Kyoto Prefectural University of Medicine, Kyoto, Japan; b Department of Medical Oncology, Fukuchiyama City Hospital, Kyoto, Japan; c Division of Respirology, Neurology, and Rheumatology, Department of Internal Medicine, Kurume University School of Medicine, Fukuoka City, Japan; d Respiratory Center, Matsusaka Municipal Hospital Respiratory Center, Matsusaka, Japan; e Department of Respiratory Medicine, Fujita Health University of Medicine, Aichi, Japan; f Respiratory Medicine, Kanazawa University Hospital, Ishikawa, Japan; g Department of Respiratory Medicine, Japanese Red Cross Kyoto Daiichi Hospital, Kyoto, Japan; h Department of Pulmonary Medicine, Rakuwakai Otowa Hospital, Kyoto, Japan; i Department of Respiratory Medicine, Uji-Tokushukai Medical Center, Kyoto, Japan; j Department of Respiratory Medicine, Japanese Red Cross Kyoto Daini Hospital, Kyoto, Japan; k Department of Pulmonary Medicine, Otsu City Hospital, Shiga, Japan; l Department of Pulmonary Medicine, Saiseikai Shiga Hospital, Shiga, Japan

**Keywords:** Immune checkpoint inhibitor, Non–small cell lung cancer, Osimertinib, *EGFR* mutation, Chemoimmunotherapy

## Abstract

**Introduction:**

The use of immune checkpoint inhibitors (ICIs) with chemotherapy has increased the survival of patients with advanced NSCLC. Nevertheless, the efficacy of ICI treatment for NSCLC with *EGFR* mutations is limited. Previous studies have not evaluated the efficacy of ICI treatment after osimertinib treatment in real-world settings.

**Methods:**

This study performed a retrospective analysis of the association between clinical characteristics and ICI efficacy in patients with *EGFR*-mutant NSCLC treated with ICIs after osimertinib treatment at 12 institutions in Japan from March 2016 to March 2021.

**Results:**

Among 80 patients with *EGFR*-mutant lung cancer, 42 received ICI monotherapy and 38 received chemoimmunotherapy. In the chemoimmunotherapy group, the progression-free survival (PFS) was significantly longer in the group that exhibited PFS more than 10 months with osimertinib than in the group that exhibited PFS less than or equal to 10 months with osimertinib (8.4 mo versus 3.8 mo, *p* = 0.026). Nevertheless, there was no significant difference in PFS in the ICI monotherapy group (1.7 mo versus 1.5 mo, *p* = 0.45). Regardless of the *EGFR* mutation subtype, PFS of osimertinib treatment was a predictor of the PFS of chemoimmunotherapy (exon 19 deletion mutation: *p* = 0.03 and exon 21 L858R mutation: *p* = 0.001).

**Conclusions:**

The PFS of osimertinib might be a predictor of PFS of chemoimmunotherapy in patients with *EGFR*-mutant NSCLC. Further clinical investigations on the predictors of efficacy of administering ICIs after osimertinib treatment are required.

## Introduction

Immune checkpoint inhibitors (ICIs) have been approved to treat various carcinoma types, including lung cancer.^1^, ^2^, ^3^ ICIs exert their antitumor effects by inhibiting the immune escape mechanism from immune cell attacks. These effects of ICIs are mediated by the binding inhibition of programmed cell death protein 1 to programmed death-ligand 1(PD-L1). This results in the activation of cancer antigen-specific T-cells and enhancement of cytotoxic activity.^4^

ICIs are frequently used for the treatment of lung cancer. Nevertheless, ICI efficacy in patients with lung cancer harboring *EGFR* mutations is limited on the basis of prospective trials and a registry trial.^5^^,^^6^ In contrast, a clinical trial of chemotherapy with ICIs, including atezolizumab, in NSCLC reported that the combination has therapeutic efficacy in a subgroup analysis of patients with *EGFR*-mutant NSCLC.^7^ Therefore, the clinical impact of ICIs in patients with *EGFR*-mutant NSCLC is not fully understood. In addition, evidence of its efficacy in patients with lung cancer and *EGFR* mutations was based on treatment with first- or second-generation *EGFR* tyrosine kinase inhibitor (TKI).

Osimertinib is a third-generation *EGFR* TKI approved in several countries to treat *EGFR*-T790M mutation-positive unresectable or recurrent NSCLC that is refractory to first- and second-generation *EGFR* TKIs.^8^^,^^9^ Osimertinib was found to have a marked therapeutic effect in the FLAURA trial compared with first-generation *EGFR* TKIs as a first-line treatment for *EGFR*-mutant lung cancer.^10^^,^^11^ Although osimertinib is frequently used for its potential beneficial outcomes in first- and late-line settings, almost all patients with *EGFR*-mutant lung cancer ultimately acquired resistance to osimertinib after approximately 20 months.^10^ Moreover, little is known about the efficacy of ICI-containing regimens after osimertinib treatment.

To investigate this topic in real-world settings, the efficacy of ICIs in patients with *EGFR*-mutant NSCLC treated with osimertinib at 12 different institutions in Japan was retrospectively analyzed.

## Materials and Methods

### Methods

The medical records of consecutive patients with *EGFR*-mutant NSCLC who received ICIs after osimertinib were screened from March 2016 to March 2021. Of these patients, the data of those who met the registration criteria were obtained from 12 institutions in Japan. The criteria for inclusion were as follows: patient aged more than or equal to 20 years; histologically confirmed NSCLC (classified based on response evaluation criteria in solid tumors, version 1.1 criteria for measurable disease); confirmed *EGFR*-activating mutation (including one or more of the following: *EGFR* exon 19 deletion, S768I, L858R, L861Q, and G719X); and received osimertinib treatment, ICI monotherapy, or chemoimmunotherapy in posterior lines. Treatment decisions were made at the attending physician’s discretion on the basis of the patient's conditions. The data cutoff date was August 31, 2021. The primary end point was progression-free survival (PFS) to ICIs. The secondary end point was the association of PFS with osimertinib and ICIs. Patients were divided into two groups on the basis of PFS to osimertinib, and the PFS with ICIs was compared. A cutoff of 10 months for PFS with osimertinib was set, considering the cutoff values of previous studies.^12^^,^^13^ Patients who discontinued osimertinib owing to adverse events before progression were excluded from PFS analysis.

Data on patient characteristics such as age, sex, histologic type, PD-L1 expression, *EGFR* gene mutation status, Eastern Cooperative Oncology Group (ECOG) performance status (PS), smoking history, PFS to osimertinib and ICI therapy, overall survival (OS), objective response rate (ORR), and disease control rate were retrieved from medical records. ECOG-PS scores and age were evaluated at the start of ICI treatment. The eighth edition of the American Joint Commission on Cancer staging system was used to assess staging. Tumor response was determined on the basis of the Response Evaluation Criteria in Solid Tumors, version 1.1.

This study was approved by the Ethics Review Board of the Kyoto Prefectural University of Medicine and was conducted with the approval of the Ethics Review Board of each hospital (approval number ERB-C-1918).

### Assessment of Efficacy

PFS was defined as the time from the first administration of osimertinib or ICIs to disease progression or death. The cutoff was the next treatment start date if the treatment was changed owing to adverse events or other reasons before disease progression. OS is the time from the first administration of ICIs to death. PFS and OS were censored on the final confirmation of the survival of patients whose disease did not progress and those who survived.

### Tumor Genetic Analysis

*EGFR* mutations were detected by either peptide nucleic acid lock nucleic acid clamp (LSI Medience, Tokyo, Japan), cycleave polymerase chain reaction (Takara Bio, Kusatsu, Japan), or Cobas *EGFR* mutation test (Roche Molecular Systems, Pleasanton, CA). Sequencing of exons 18 to 21 was performed by commercial clinical laboratories (SRL Inc. and BML Inc., Tokyo, Japan). Uncommon mutations were defined as mutations other than the 19 deletions and the L858R mutation.

### PD-L1 Analysis of Tumor

The 22C3 antibody (Agilent Technologies, Santa Clara, CA) measured tumor PD-L1 expression. PD-L1 expression was measured using tissue samples at the time of diagnosis.

### Statistical Analysis

Fisher’s exact test or the chi-square test was performed for categorical variables. The Mann-Whitney *U* test compared the number of treatment lines for ICI-based therapy. PFS and OS were evaluated using the Kaplan-Meier method, and differences were compared using the log-rank test. In the univariate and multivariate analyses, a Cox proportional hazard model was used to estimate hazard ratios (HRs) and 95% confidence intervals (CIs). On the basis of previous reports, sex, age (≥70 y), ECOG PS (PS ≥ 2), smoking status, uncommon *EGFR* mutations, and PFS after osimertinib treatment for more than 10 months were selected as covariates.^12^ No patient had uncommon mutations in the ICI monotherapy group; therefore, this factor was excluded from the covariates. We set statistical significance at *p* value less than 0.05. The analyses were performed with software EZR (version 1.54).^14^

## Results

### Patients’ Characteristics

A total of 80 patients from 12 institutions in Japan between March 2016 and March 2021 were enrolled. The median PFS of osimertinib treatment was 8.5 months (95% CI: 6.8–11.0 mo) (Supplementary Fig. 1*A*). There was no significant difference in PFS of osimertinib between patients who received first-line treatment with osimertinib and those who received osimertinib after second-line or later treatment (9.8 versus 8.3 mo; log-rank test, *p* = 0.77) (Supplementary Fig. 1*B*). In addition, there was no significant difference in PFS of osimertinib between patients who had exon 19 deletions and those who had exon 21 L858R mutation (9.2 versus 9.8 mo; log-rank test, *p* = 0.69) (Supplementary Fig. 1*C*). Of the enrolled patients, 42 who received ICI monotherapy and 38 who received chemoimmunotherapy were evaluated separately, as illustrated in Table 1. The median follow-up time for censored cases was 25.6 and 15.3 months in the ICI monotherapy regimen and the chemoimmunotherapy regimen groups, respectively. In the ICI monotherapy regimen group, the median age was 68 (range: 43–85) years, 21 patients (50.0%) were of male sex, 14 (33.3%) had ECOG PS 2/3, 16 (38.1%) had a history of smoking, and nine (21.4%) had a PD-L1 tumor proportion score more than or equal to 50%. In the chemoimmunotherapy regimen group, the median age was 66 (range: 39–79) years, 22 patients (57.9%) were of male sex, five (13.2%) had ECOG PS 2/3, three (7.9%) had uncommon *EGFR* mutations, 19 (50.0%) had a history of smoking, and nine (23.7%) had a PD-L1 tumor proportion score greater than or equal to 50%. Furthermore, 28 patients received carboplatin plus paclitaxel plus atezolizumab plus bevacizumab. Another chemoimmunotherapy regimen for 10 patients was as follows: one patient with carboplatin plus etoposide plus atezolizumab, one with carboplatin plus nab-paclitaxel plus atezolizumab, five with carboplatin plus pemetrexed plus atezolizumab, and three with carboplatin plus pemetrexed plus pembrolizumab.

**Table 1:** Patient Characteristics

Characteristics	All Patients (N = 80)	ICI Monotherapy (n = 42)	Chemoimmunotherapy (n = 38)	*p* Value
Age				
Median (range)	68 (39–85)	68 (43–85)	66 (39–79)	0.15
Sex, n (%)				
Male	43 (53.8)	21 (50.0)	22 (57.9)	0.51
Female	37 (46.2)	21 (50.0)	16 (42.1)	
ECOG performance status, n (%)				
0	11 (13.8)	3 (7.1)	8 (21.1)	0.04^a^
1	50 (62.5)	25 (59.5)	25 (65.8)	
2/3	19 (23.8)	14 (33.3)	5 (13.2)	
Stage, n (%)				
Postoperative recurrence	11 (13.8)	5 (11.9)	6 (15.8)	0.75
Ⅲ	5 (6.2)	3 (7.1)	2 (5.3)	
IV	64 (80.0)	34 (81.0)	30 (78.9)	
*EGFR* mutation, n (%)				
19 deletion	44 (55.0)	26 (61.9)	18 (47.4)	0.10^b^
L858R	33 (41.3)	16 (38.1)	17 (44.7)	
G719X	3 (3.7)	0 (0)	3 (7.9)	
Smoking status, n (%)				
Current/former	35 (43.8)	16 (38.1)	19 (50.0)	0.37
Never	45 (56.2)	26 (61.9)	19 (50.0)	
Histology, n (%)				
Adenocarcinoma	79 (98.7)	41 (97.6)	38 (100.0)	1.0
Squamous cell carcinoma	1 (1.3)	1 (2.4)	0 (0)	
PD-L1 TPS, n (%)				
≥50%	18 (22.5)	9 (21.4)	9 (23.7)	1.0^c^
1%–49%	22 (27.5)	8 (19.0)	14 (36.8)	
<1%	19 (23.8)	12 (28.6)	7 (18.4)	
Unknown	21 (26.3)	13 (31.0)	8 (21.1)	
Treatment line of osimertinib, n (%)				
First line	33 (41.3)	9 (21.4)	24 (63.2)	0.001
Second line or later (T790M positive)	47 (58.7)	33 (78.6)	14 (36.8)	
Treatment line of ICI-based therapy				
Median (range)	3 (2–14)	5 (3–14)	3 (2–5)	<0.001^d^
Agents immediately before ICI-based therapy, n (%)				
Osimertinib	48 (60.0)	17 (40.5)	31 (81.6)	<0.001
Others	32 (40.0)	25 (59.5)	7 (18.4)	
Median PFS of osimertinib				
Month (95% confidence interval)	8.5 (6.8–11.0)	8.3 (4.7–10.9)	9.8 (6.5–11.7)	0.36^e^

ECOG, Eastern Cooperative Oncology Group; ICI, immune checkpoint inhibitor; PD-L1 TPS, programmed death-ligand 1 tumor proportion score; PFS, progression-free survival.

a Performance status 0/1 versus 2/3.

b *EGFR* mutation uncommon versus common mutation.

c PD-L1 TPS ≥ 50% versus all others except for unknown.

d Calculated with Mann-Whitney *U* test.

e Calculated with log-rank test.

### Treatment Efficacy of ICI-Based Regimen in Patients With EGFR-Mutant Lung Cancer

**Figure 1:**
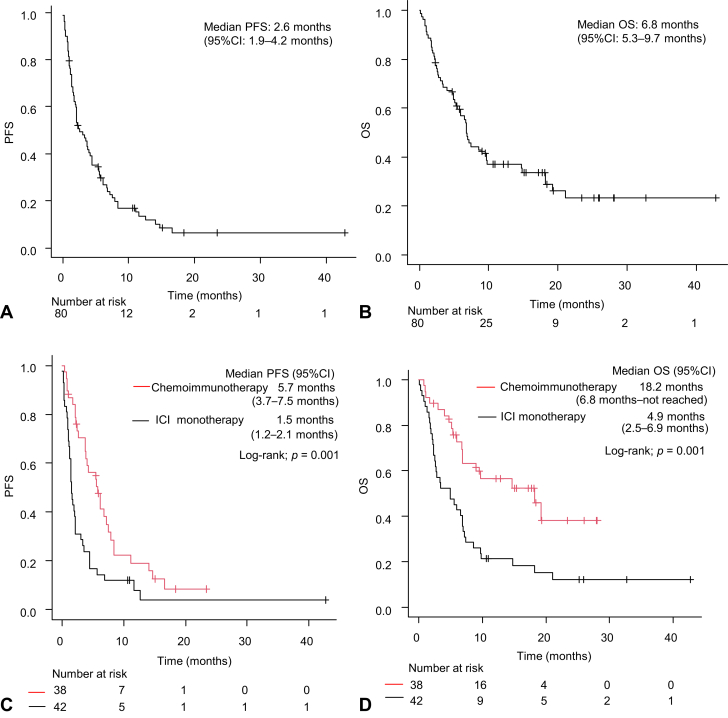
(*A*) PFS and (*B*) OS of ICI-based therapy in all patients (N = 80). (*C*) PFS and (*D*) OS of ICI-based therapy according to the treatment regimen. CI, confidence interval; HR, hazard ratio; ICI, immune checkpoint inhibitor; OS, overall survival; PFS, progression-free survival.

The ORR of patients with *EGFR*-mutant lung cancer who received an ICI-based regimen was 16.3% (Supplementary Fig. 2*A*). The median PFS with ICI-based regimen was 2.6 months (95% CI: 1.9–4.2 mo) (Fig. 1*A*). The median OS with ICI-based regimen was 6.8 months (95% CI: 5.3–9.7 mo) (Fig. 1*B*). The ORR of the ICI monotherapy regimen group was 10.5% (Supplementary Fig. 2*B*), and their median PFS was 1.5 months (95% CI: 1.2–2.1 mo) (Fig. 1*C*). The ORR of the chemoimmunotherapy regimen group was 23.7% (Supplementary Fig. 2*C*), and the median PFS was 5.7 months (95% CI: 3.7–7.5 mo) (Fig. 1*C*). The median PFS and OS were significantly longer with the chemoimmunotherapy regimen than with the ICI monotherapy regimen (5.7 versus 1.5 mo, log-rank test *p* = 0.001, and 18.2 versus 4.9 mo, log-rank test *p* = 0.001, respectively) (Fig. 1*C* and *D*).

### Association Between Clinicopathologic Factors and ICI-Based Regimen

The association between clinicopathologic factors and PFS of ICI-based regimens was investigated to determine the characteristics of patients who benefited from ICI-based therapy. In the chemoimmunotherapy group, patients with a PFS of more than 10 months with osimertinib had a higher PFS with chemoimmunotherapy (HR = 0.23, 95% CI: 0.09–0.59, *p* = 0.002) (Tables 2 and 3). Univariate and multivariate analyses in the ICI monotherapy group revealed that patients with PS greater than or equal to 2 had a significantly shorter PFS than those with PS 0/1 (HR = 2.32, 95% CI: 1.14–4.73, *p* = 0.02). Multivariate analysis in the chemoimmunotherapy group revealed that patients with uncommon mutations had significantly better PFS than those with common mutations (HR = 0.17, 95% CI: 0.03–0.96, *p* = 0.04). In addition, the association between clinicopathologic factors and OS with ICI-based regimens was investigated. In the chemoimmunotherapy group, consistent with earlier PFS analysis, patients with longer PFS of osimertinib had longer OS, although not significant (HR = 0.67, 95% CI: 0.23–1.92, *p* = 0.45) (Supplementary Tables 1 and 2). In the ICI monotherapy group, patients with PS greater than or equal to 2 had a significantly shorter OS than those with PS 0/1 (HR = 5.83, 95% CI: 2.44–13.9, *p* < 0.001) (Supplementary Table 2).

**Table 2:** Cox Proportional Hazard Models for Time to PFS in Patients With NSCLC Harboring *EGFR* Who Received ICI-Based Therapy

Items	ICI Monotherapy			Chemoimmunotherapy		
	Patient’s No.	PFS (mo)		Patient’s No.	PFS (mo)	
		Median PFS (95% CI)	*p* Value		Median PFS (95% CI)	*p* Value
Age, y						
<70	16	1.3 (0.3–3.5)	0.40	21	3.7 (2.1–7.2)	0.10
≥70	26	1.7 (1.2–2.1)		17	6.7 (4.0–14.1)	
Sex						
Male	21	1.5 (1.1–3.5)	0.47	22	3.8 (1.7–7.2)	0.18
Female	21	1.5 (0.9–2.1)		16	6.1 (4.0–8.4)	
ECOG PS						
0/1	28	2.0 (1.4–3.5)	0.04	33	6.1 (3.7–7.9)	0.26
≥2	14	1.0 (0.2–1.7)		5	2.6 (0.8–NA)	
Stage						
Postoperative recurrence	5	2.1 (1.2–NA)	0.33	6	8.4 (6.1–NA)	0.15
Ⅲ/IV	37	1.4 (1.0–2.1)		32	4.2 (2.4–6.1)	
*EGFR* mutation						
Common mutation	42	1.5 (1.2–2.1)	NA	35	5.4 (2.6–7.2)	0.26
Uncommon mutation	0	NA		3	7.9 (5.5–NA)	
PD-L1 expression						
≥50%	9	1.6 (0–NA)	0.40	9	5.5 (0.4–16.6)	0.22
<50%	20	1.7 (1.1–3.3)		21	4.0 (2.1–6.7)	
Histology						
Adenocarcinoma	41	1.5 (1.1–2.1)	NA	38	5.7 (3.7–7.5)	NA
Squamous cell carcinoma	1	NA		0	NA	
Smoking history						
Current/former	16	2.0 (1.2–4.4)	0.26	19	3.7 (1.0–6.1)	0.28
Never	26	1.5 (0.9–2.0)		19	6.7 (4.0–8.4)	
PFS of osimertinib						
>10 mo	15	1.7 (1.0–4.4)	0.47	17	8.4 (5.4–14.1)	0.03
≤10 mo	24	1.5 (0.8–2.1)		18	3.8 (2.1–5.7)	
Treatment line of osimertinib						
First line	9	1.1 (0–4.4)	0.58	24	5.4 (2.6–7.9)	0.51
Second line or later (T790M positive)	33	1.6 (1.2–2.1)		14	6.1 (2.1–8.4)	
Reason for osimertinib discontinuation						
Progressive disease	39	1.5 (1.2–2.1)	0.27	35	6.1 (3.7–7.5)	0.08
Adverse event	3	0.9 (0.7–NA)		3	3.7 (0.4–NA)	
Antiangiogenesis						
With bevacizumab		NA	NA	28	5.5 (2.6–7.5)	0.62
Without bevacizumab		NA		10	7.0 (0.8–14.1)	

CI, confidence interval; ECOG, Eastern Cooperative Oncology Group; ICI, immune checkpoint inhibitor; NA, not available; PD-L1, programmed death-ligand 1; PFS, progression-free survival.

**Table 3:** Cox Proportional Hazard Models for PFS in Patients With NSCLC Harboring *EGFR* Mutation Who Received ICI-Based Therapy in Multivariate Analysis

Items	ICI Monotherapy		Chemoimmunotherapy	
	PFS (Multivariate Analysis)		PFS (Multivariate Analysis)	
	HR (95% CI)	*p* Value	HR (95% CI)	*p* Value
Age ≥ 70 y	0.77 (0.38–1.56)	0.46	0.46 (0.20–1.02)	0.06
Female sex	0.91 (0.35–2.37)	0.85	1.24 (0.49–3.14)	0.66
ECOG-PS ≥ 2	2.32 (1.14–4.73)	0.02	0.98 (0.30–3.23)	0.98
Smoking history	0.61 (0.22–1.66)	0.33	1.06 (0.43–2.65)	0.86
*EGFR* uncommon mutation^a^	NA	NA	0.17 (0.03–0.96)	0.04
PFS of osimertinib > 10 mo	0.77 (0.38–1.55)	0.46	0.23 (0.09–0.59)	0.002

CI, confidence interval; ECOG PS, Eastern Cooperative Oncology Group performance status; HR, hazard ratio; NA, not available; PFS, progression-free survival.

a *EGFR* mutation uncommon versus common mutation.

### Association Between PFS of Osimertinib Treatment and ICI-Based Regimen

**Figure 2:**
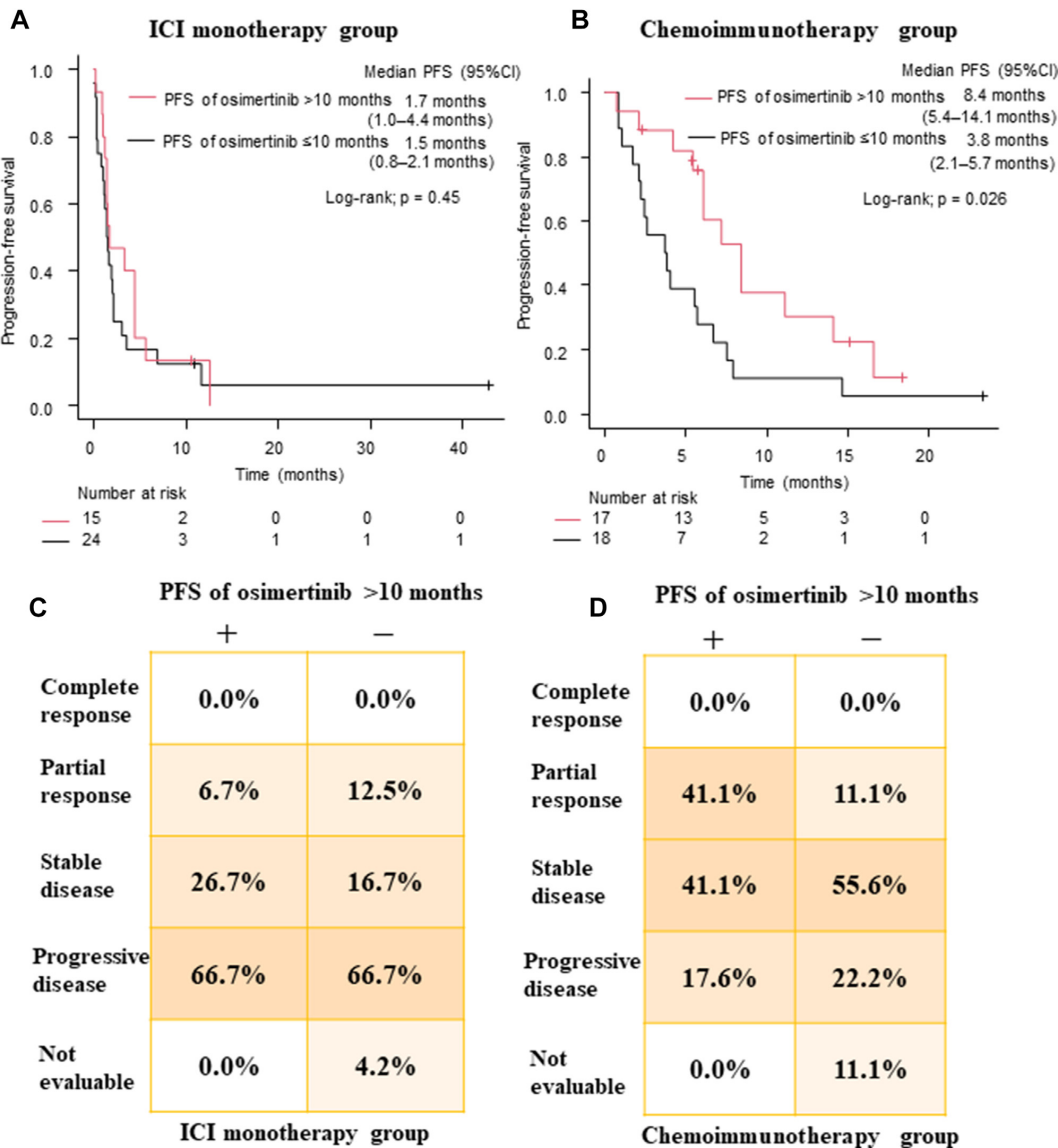
(*A*) PFS of ICI monotherapy in patients who responded to osimertinib more than 10 months (red line) and those who responded to osimertinib less than or equal to 10 months (black line). (*B*) PFS of chemoimmunotherapy in patients who responded to osimertinib more than 10 months (red line) and those who responded to osimertinib less than or equal to 10 months (black line). (*C*) Comparison of treatment responses between patients who responded to osimertinib more than 10 months and those who responded to osimertinib less than or equal to 10 months in the ICI monotherapy group. (*D*) Comparison of treatment response between patients who responded to osimertinib more than 10 months and those who responded to osimertinib less than or equal to 10 months in the chemoimmunotherapy group. CI, confidence interval; HR, hazard ratio; ICI, immune checkpoint inhibitor; PFS, progression-free survival.

Kaplan-Meier plots divided by PFS of osimertinib treatment overlapped in the ICI monotherapy group but were clearly separated in the chemoimmunotherapy group (ICI monotherapy group, median PFS in the group with PFS > 10 mo with osimertinib: 1.7 mo, 95% CI: 1.0–4.4 mo, and group with PFS ≤ 10 mo with osimertinib: 1.5 mo, 95% CI: 0.8–2.1 mo, log-rank test, *p* = 0.45; chemoimmunotherapy group, median PFS in the group with PFS > 10 mo with osimertinib: 8.4 mo, 95% CI: 5.4–14.1 mo, and group with PFS ≤ 10 mo osimertinib: 3.8 mo, 95% CI: 2.1–5.7 mo, log-rank test, *p* = 0.026) (Fig. 2*A* and *B*). The ORR was higher in patients with a PFS more than 10 months with osimertinib than in those with less than or equal to 10 months in the chemoimmunotherapy group (41.1% versus 11.1%, *p* = 0.06) (Fig. 2*D*). In contrast, such a significant difference was not observed in the ORR of the ICI monotherapy group (6.7% versus 12.5%, *p* = 1.0) (Fig. 2*C*).

### Impact of PFS With Osimertinib According to EGFR Mutation Status in Chemoimmunotherapy Group

**Figure 3:**
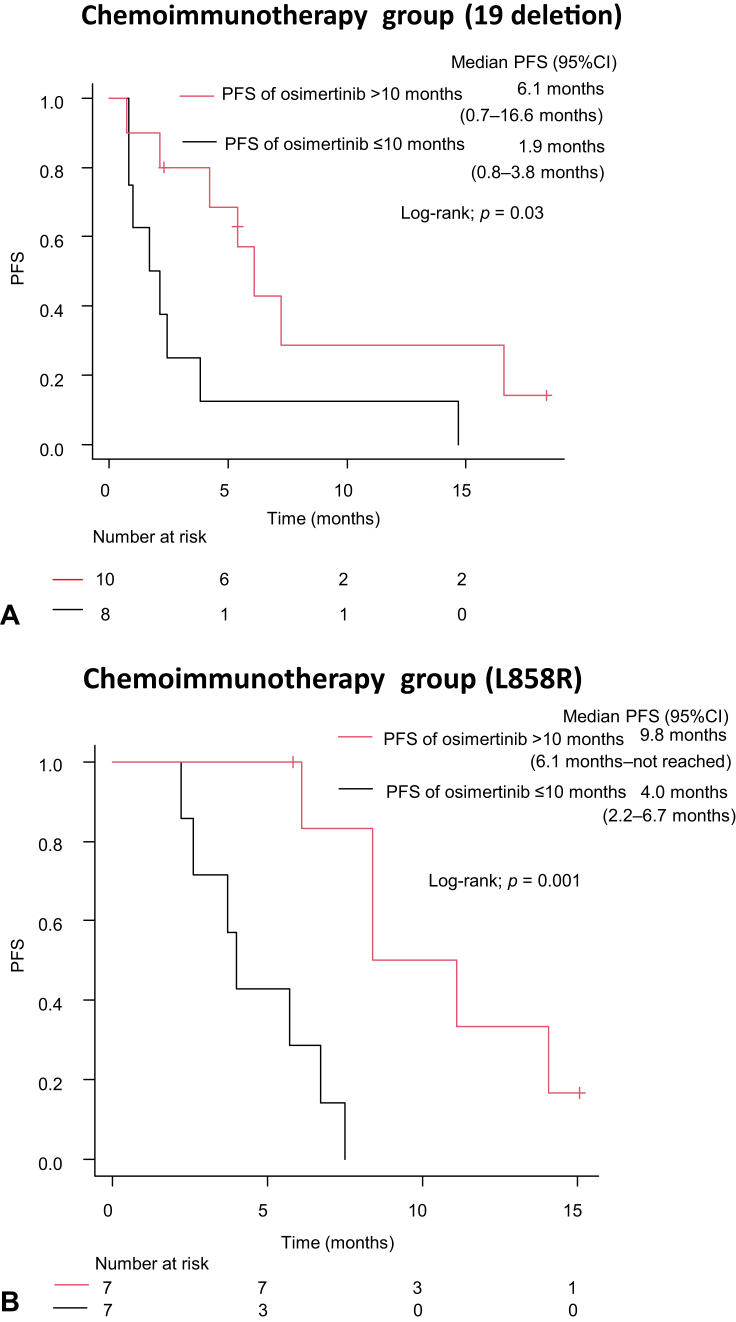
PFS of chemoimmunotherapy in patients with NSCLC who had (*A*) 19 deletion and (*B*) L858R mutation according to PFS of osimertinib (cutoff 10 mo). Patients who discontinued osimertinib owing to adverse events were excluded from the analysis. CI, confidence interval; HR, hazard ratio; OS, overall survival; PFS, progression-free survival.

We investigated the correlation between PFS with osimertinib and PFS with chemoimmunotherapy in patients with *EGFR*-mutant NSCLC with exon 19 deletions and L858R mutations. There was no significant difference in PFS with chemoimmunotherapy between patients with NSCLC with exon 19 deletion mutation and those with L858R mutation (3.8 mo, 95% CI: 1.7–7.2 mo versus 6.1 mo, 95% CI: 3.7–8.4 mo; log-rank test, *p* = 0.57) (Supplementary Fig. 3*C*). Patients with NSCLC who received chemoimmunotherapy were divided into two groups according to their *EGFR* mutation status. In patients with NSCLC with exon 19 deletion, PFS with chemoimmunotherapy was significantly longer in the group with PFS more than 10 months with osimertinib than in the group with less than or equal to 10 months (6.1 mo, 95% CI: 0.7–16.6 mo versus 1.9 mo, 95% CI: 0.8–3.8 mo; log-rank test, *p* = 0.03) (Fig. 3*A*). Furthermore, patients with NSCLC with L858R mutation also had significantly longer PFS with chemoimmunotherapy in the group with PFS more than 10 months with osimertinib than in the group with less than or equal to 10 months (9.8 mo, 95% CI: 6.1 mo–not reached versus 4.0 mo, 95% CI: 2.2–6.7 mo; log-rank test, *p* = 0.001) (Fig. 3*B*).

## Discussion

Several previous studies have investigated the clinical impact of ICIs after first- and second-generation *EGFR* TKIs in patients with *EGFR*-mutant NSCLC. Moreover, the association between PFS of first- and second-generation *EGFR* TKIs and PFS of ICIs remains controversial.^12^^,^^13^^,^^15^ In contrast, we evaluated the efficacy of ICI-based therapy after osimertinib treatment, including its correlation with the efficacy of osimertinib. This study revealed that a longer PFS of osimertinib might predict outcomes of chemoimmunotherapy treatment but not ICI monotherapy in patients with *EGFR*-mutant NSCLC. This suggests that clinical outcomes of osimertinib might be meaningful for predicting those of chemoimmunotherapy.

Long-term *EGFR* TKI administration has been suggested to induce an intrinsic interferon response in tumor cells, which may improve the tumor microenvironment (TME) by increasing T-cell infiltration that contributes to the treatment response.^16^ Furthermore, a previous report revealed that the intervention of *EGFR* TKI decreased CD4+ effector regulatory T-cell infiltration in the TME, implying it might improve the efficacy of immunotherapy.^17^ These findings suggest the possibility of clinical benefit with subsequent ICI-based therapy for patients with *EGFR*-mutated NSCLC after acquired resistance to osimertinib. Nevertheless, the therapeutic effect of ICI monotherapy is generally poor in patients with *EGFR*-mutant NSCLC.^5^^,^^6^ In our study, the median PFS of ICI monotherapy was 1.5 months, which is poor, similar to previous reports.

It was reported that disease progression after *EGFR* TKI treatment leads to suppression of tumor-infiltrating CD8+ T cells and induction of regulatory T cells, resulting in a noninflamed TME and insensitivity to ICI monotherapy.^18^ In contrast, chemotherapeutic agents have been found to enhance CD8+ T cell infiltration and deplete immunosuppressive cells.^19^^,^^20^ Therefore, the use of chemoimmunotherapy, a combination of ICIs and cytotoxic anticancer drugs, provides an improved immunologic status in the TME and theoretically facilitates an antitumor immune response in tumors compared with that of ICI monotherapy.^19^ In this study, the effect of the chemotherapeutic agents’ combination might have been stronger in the group that had a longer PFS with osimertinib treatment. Further investigations are required to confirm the response to chemoimmunotherapy in patients with *EGFR*-mutant NSCLC after osimertinib treatment.

In multivariate analysis, patients with uncommon *EGFR* mutations had a significantly better PFS with chemoimmunotherapy than those with common *EGFR* mutations. Our previous study revealed that patients with uncommon mutations had significantly prolonged PFS of ICI monotherapy compared with patients with common mutations.^21^ These patients may benefit from chemoimmunotherapy. Nevertheless, the number of cases was minimal and only patients with G719X were included. Therefore, further large-scale cohort investigations are required.

This study revealed that a longer PFS with osimertinib treatment was associated with a longer PFS with chemoimmunotherapy, regardless of the *EGFR* mutation subtype. Hastings et al.^22^ reported that patients with NSCLC with exon 19 deletion mutations had a lower tumor mutation burden and poorer response to ICIs than those with the L858R mutation. This result suggests that *EGFR* mutation subtypes in NSCLC may affect the efficacy of ICIs and correlate with the tumor mutation burden. In contrast, there was no significant difference in the efficacy of chemoimmunotherapy in this study according to the subtype of *EGFR* mutation, which may be due to the small sample size for the subanalysis (n = 36) and the lack of statistical power.

This study had some limitations. First, although this study included patients from several institutions, the number remained moderate. Second, platinum-containing cytotoxic chemotherapy was not compared; therefore, optimal chemotherapy after osimertinib administration was not determined. Third, many factors can influence clinical outcomes after osimertinib treatment and not all factors can be adjusted for. Fourth, in this study, the median follow-up time was 15.3 months in the chemoimmunotherapy group, which had many censored cases. We consider that the short follow-up period may have affected the inconclusive results of OS. Finally, patients who received chemoimmunotherapy had better general conditions (e.g., good PS) than those who received ICI monotherapy. Intrinsic differences between the two groups may have affected the results.

In conclusion, a longer PFS of osimertinib might be associated with a longer PFS of chemoimmunotherapy in patients with NSCLC with *EGFR* mutations. Further studies, including a comparison of platinum-based cytotoxic chemotherapy with other chemotherapeutic agents, are needed to evaluate the efficacy of ICIs in patients with *EGFR*-mutant NSCLC.

## CRediT Authorship Contribution Statement

**Kenji Morimoto, Ryo Sawada, Tadaaki Yamada, Koichi Takayama:** Study conception, Design.

**Kenji Morimoto, Ryo Sawada, Koichi Azuma, Kentaro Ito, Yasuhiro Goto, Hideharu Kimura, Taishi Harada, Shinsuke Shiotsu, Nobuyo Tamiya, Yusuke Chihara, Takayuki Takeda, Osamu Hiranuma, Isao Hasegawa:** Obtain clinical data.

**Kenji Morimoto, Ryo Sawada, Tadaaki Yamada, Yoshie Morimoto, Masahiro Iwasaku, Shinsaku Tokuda, Koichi Takayama:** Data interpretation, Manuscript preparation.

**Kenji Morimoto, Ryo Sawada, Tadaaki Yamada, Koichi Azuma, Kentaro Ito, Yasuhiro Goto, Hideharu Kimura, Taishi Harada, Shinsuke Shiotsu, Nobuyo Tamiya, Yusuke Chihara, Takayuki Takeda, Osamu Hiranuma, Isao Hasegawa, Yoshie Morimoto, Masahiro Iwasaku, Shinsaku Tokuda, Koichi Takayama:** Read and Approval of final version of the manuscript.

## Ethics Approval and Consent to Participate

The study protocol was approved by the ethics committee of each hospital, including the Kyoto Prefectural University of Medicine (approval no. ERB-C-1918). Because this was a retrospective study, the need for informed consent was waived, and an official website was used as an opt-out method, which was approved by the ethics committee of each hospital.

## Availability of Data and Material

The data sets generated during the current study are not publicly available because of ethical constraints but are available from the corresponding author on reasonable request.
