# Supplementary material for: A Real-World Analysis of Immune Checkpoint Inhibitor-Based Therapy After Osimertinib Treatment in Patients With EGFR-Mutant NSCLC
Source: JTO Clin Res Rep. 2022 Aug 6;3(9):100388. doi: 10.1016/j.jtocrr.2022.100388 (PMC9445370; doi:10.1016/j.jtocrr.2022.100388)
Supplement: Supplementary Figure [file mmc1.pdf]

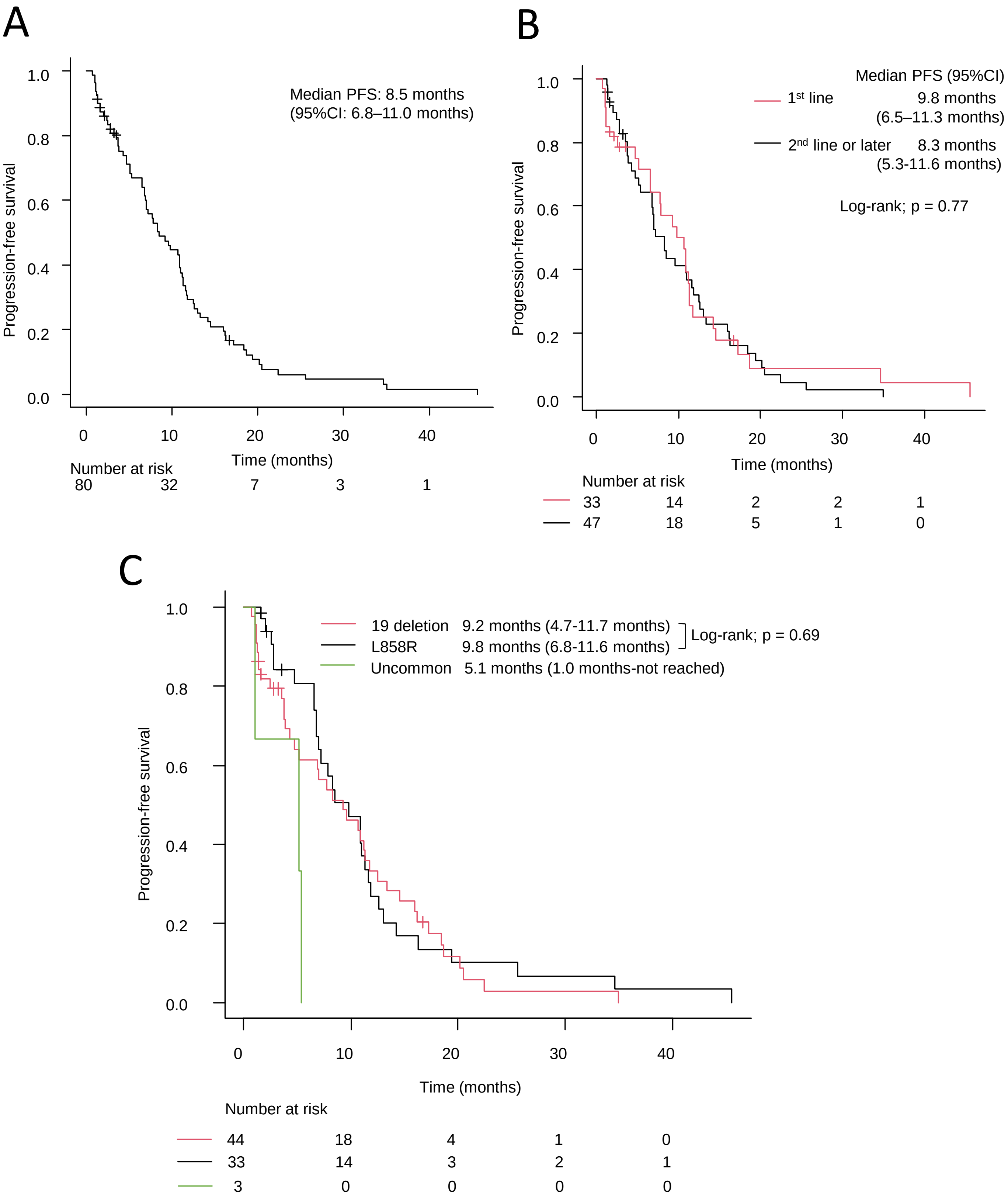

Supplementary Fig. 1

(A) PFS of osimertinib in all patients (N = 80). (B) PFS of osimertinib in NSCLC patients who received first line osimertinib (red line) and second line or later osimertinib (black line). (C) PFS of osimertinib in NSCLC patients who had exon 19 deletion (red line), L858R mutation(black line), and uncommon mutation (green line).

PFS, progression-free survival; NSCLC, non-small cell lung cancer; CI, confidence interval.

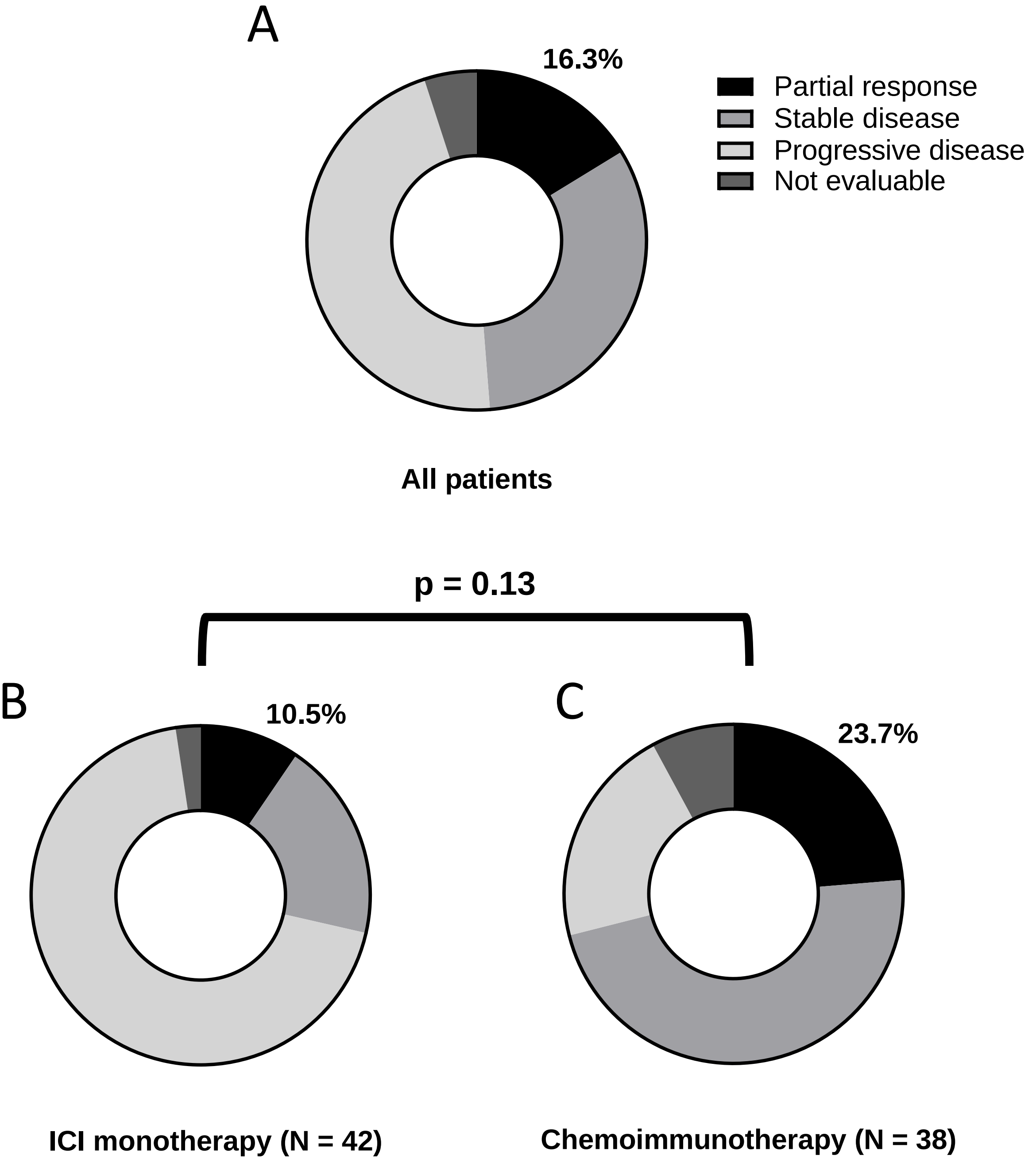

Supplementary Figure 2

Best overall response to ICI based therapy after osimertinib treatment for all patients (A),

, ICI monotherapy (B), and chemoimmunotherapy (C).

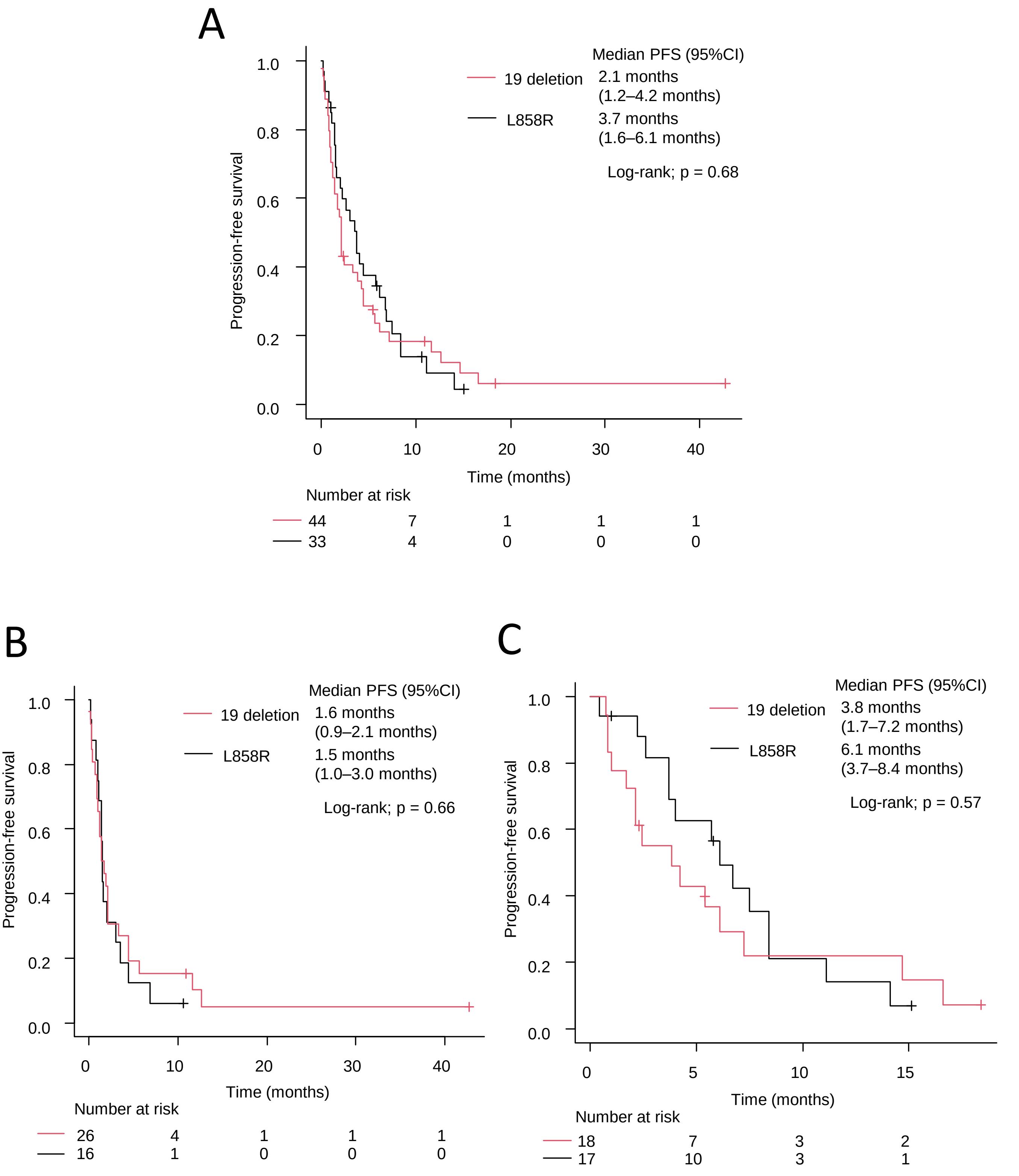

Supplementary Figure 3

(A) PFS of ICIs in all patients except for uncommon mutations (N=77) who had exon 19 deletion (red line) and L858R mutation (black line). PFS of (B) ICI monotherapy and (C) chemoimmunotherapy in NSCLC patients who had exon 19 deletion (red line) and L858R mutation (black line).

ICI, immune-checkpoint inhibitor; NSCLC, non-small cell lung cancer; HR, hazard ratio; CI, confidence interval.
