# Supplementary material for: A Real-World Analysis of Immune Checkpoint Inhibitor-Based Therapy After Osimertinib Treatment in Patients With EGFR-Mutant NSCLC
Source: JTO Clin Res Rep. 2022 Aug 6;3(9):100388. doi: 10.1016/j.jtocrr.2022.100388 (PMC9445370; doi:10.1016/j.jtocrr.2022.100388)
Supplement: Supplementary Table [file mmc2.pdf]

Supplementary Table 1. Cox proportional-hazards models for time to overall survival in patients with non-small cell lung cancer harboring EGFR who received ICI based therapy

Items	ICI monotherapy			Chemoimmunotherapy		
	Patient's No.	OS (months)		Patient's No.	OS (months)	
		Median OS (95% CI)	p-Value		Median OS (95% CI)	p-Value
Age						
<70	16	4.3 (0.8-9.7)	0.69	21	19.2 (4.7-NA)	0.51
≥70	26	4.9 (2.5-7.2)		17	18.2 (6.8-NA)	
Sex						
Male	21	3.3 (1.9-8.6)	0.84	22	9.7 (4.0-NA)	0.06
Female	21	5.9 (2.3-7.0)		16	NA (6.8-NA)	
ECOG-PS						
0/1	28	7.1 (3.4-9.8)	<0.001	33	19.2 (9.0-NA)	0.004
≥2	14	2.1 (0.5-3.3)		5	4.0 (0.8-NA)	
Stage						
Postoperative recurrence	5	6.5 (1.9-NA)	0.65	6	NA	NA
III/IV	37	4.9 (2.3-7.0)		32	9.7 (5.9-NA)	
EGFR mutation						
Common mutation	42	4.9 (2.5-6.9)	NA	35	14.7 (6.7-NA)	NA
Uncommon mutation	0	NA		3	NA	
PD-L1 expression						
≥50%	9	6.8 (0.1-NA)	0.60	9	NA (4.0-NA)	0.12
<50%	20	5.7 (2.2-8.6)		21	6.8 (4.7-18.2)	
Histology						
Adenocarcinoma	41	4.9 (2.3-6.9)	NA	38	18.2 (6.8-NA)	NA
Squamous cell carcionoma	1	NA		0	NA	
Smoking history						
Current/former	16	6.2 (2.2-9.8)	0.44	19	6.8 (3.0-NA)	0.01
Never	26	3.1 (2.1-6.8)		19	NA (9.0-NA)	
PFS of osimertinib						
>10 months	15	4.9 (2.1-7.0)	0.84	17	18.2 (6.8-NA)	0.28
≤10 months	24	5.7 (2.2-7.4)		18	9.0 (4.0-NA)	
Treatment line of osimertinib						
1st line	9	1.9 (0.1-NA)	0.48	24	19.2 (5.9-NA)	0.94
2nd line or later	33	5.5 (2.7-7.2)		14	18.2 (4.7-NA)	
Reason for osimertinib discontinuation						
Progressive disease	39	4.9 (2.5-7.0)	0.32	35	18.2 (6.8-NA)	0.91
Adverse event	3	1.8 (1.6-NA)		3	6.7 (6.7-NA)	
Anti-angiogenesis						
With bevacizumab		NA	NA	28	9.7 (6.7-NA)	0.96
Without bevacizumab		NA		10	18.2 (0.8-NA)	

EGFR, epidermal growth factor receptor; ECOG, eastern cooperative oncology group; ICI, immune-checkpoint inhibitor; PD-L1, programmed death ligand 1; OS, overall survival; NA, not available.

Supplementary Table 2.
Cox proportional hazard models for overall survival in patients with non-small cell lung cancer harboring EGFR mutation who received ICI based therapy in multivariate analysis.

Items	ICI monotherapy		Chemoimmunotherapy	
	OS (Multivariate analysis)		OS (Multivariate analysis)	
	HR (95% CI)	p-Value	HR (95% CI)	p-Value
Age ≥70	1.00 (0.48-2.10)	0.99	0.55 (0.19-1.64)	0.29
Female sex	0.83 (0.29-2.40)	0.74	1.20 (0.29-4.94)	0.80
ECOG-PS ≥2	5.83 (2.44-13.9)	<0.001	3.61 (0.97-13.5)	0.06
Smoking history	0.72 (0.23-2.25)	0.57	3.20 (0.84-12.2)	0.09
EGFR uncommon mutation[a]	NA	NA	NA	NA
PFS of osimertinib >10 months	1.43 (0.68-2.98)	0.35	0.67 (0.23-1.92)	0.45

[a] EGFR mutation uncommon vs. common mutation. EGFR, epidermal growth factor receptor;
ECOG-PS, eastern cooperative oncology group-performance status; CI, confidence interval; HR, hazard ratio;
OS, overall survival; NA, not available.
